# Retraction: Novel piperazine core compound induces death in human liver cancer cells: possible pharmacological properties

**DOI:** 10.1038/srep29056

**Published:** 2016-06-22

**Authors:** Nima Samie, Sekaran Muniandy, M. S. Kanthimathi, Batoul Sadat Haerian, Raja Elina Raja Azudin

Scientific Reports 6: Article number: 2417210.1038/srep24172; published online: 04132016; updated: 06222016

This Article has been retracted by the Editors and Publishers of *Scientific Reports*. Following online criticisms of the published paper, an investigation at the journal has confirmed the manipulation and duplication of data and a level of image processing that is not compliant with the journal’s policies on image data integrity in figures 1–3, 6, 7, 10 and 12. Versions of figures 1–11 and large sections of the text had also previously been published^1^,^2^.

All authors acknowledge these issues and agree to the retraction of the Article.

## References

[b1] Samie N. *et al.* Mechanism of Action of the Novel Nickel(II) Complex in Simultaneous Reactivation of the Apoptotic Signaling Networks Against Human Colon Cancer Cells. Front. Pharmacol. 6, 313 (2016). 10.3389/fphar.2015.00313 26858642 PMC4729910

[b2] Samie N., Muniandy S., Kanthimathi M. S. & Haerian B. S. Mechanism of action of novel piperazine containing a toxicant against human liver cancer cells. PeerJ 4, e1588 (2016). 10.7717/peerj.1588 27019772 PMC4806608

